# Percutaneous Nephrolithotomy Safety, Efficacy, and Outcomes: A 10-Year Experience of a Tertiary Care Center in South Lebanon

**DOI:** 10.7759/cureus.84097

**Published:** 2025-05-14

**Authors:** Alaa Jazzar, Abdallah Medlej, Nazih Rahhal, Rayan Zreik, Hassan Ali Ahmad

**Affiliations:** 1 Department of Urology, Faculty of Medicine, Lebanese University, Beirut, LBN; 2 Department of Research and Academic Affairs, Sheikh Ragheb Harb University Hospital, Nabatieh, LBN; 3 Department of Urology, Sheikh Ragheb Harb University Hospital, Nabatieh, LBN

**Keywords:** endourology, percutaneous nephrolithotomy (pcnl), renal stone disease, treatment of urolithiasis, urology surgery

## Abstract

Percutaneous nephrolithotomy (PCNL) is the gold standard for treating large and complex renal stones. This study presents a comprehensive analysis of 221 PCNL procedures performed over a decade (2014-2024) at a tertiary healthcare center in South Lebanon, focusing on safety, efficacy, and factors influencing outcomes. Patients aged 15-80 years were included, with variables such as stone characteristics, operative details, and postoperative outcomes analyzed. The mean patient age was 50 ± 16 years. The mean stone size was 3.1 ± 1.1 cm, with single stones most common (n = 82, 37.1%). The mean operative time was 2.1 ± 0.9 hours, and the average postoperative hospital stay was 2.7 ± 1.2 days. Complication rates were low (3.6%), with only one grade IV complication reported. The stone-free rate one month postoperatively was 82.35% (n = 182), with a mean residual stone size of 0.81 ± 0.32 cm, and only 8.1% (n = 18) of patients required second interventions. Regression analysis highlighted the significant influence of surgical team experience on reducing operative time, while larger stone sizes were associated with longer procedures. Compared to international benchmarks, the study demonstrated shorter operative times, lower complication rates, and comparable stone-free outcomes, emphasizing the role of expertise, advanced technologies, and structured preoperative protocols. These findings underscore the safety and efficacy of PCNL in the studied population. The study calls for the establishment of universal benchmarks for PCNL safety and efficacy, alongside multicenter studies, to optimize clinical practices and standardize outcomes for better patient care globally.

## Introduction

Percutaneous nephrolithotomy (PCNL) is a well-established, minimally invasive surgical procedure utilized to treat large, complex, or treatment-resistant kidney stones. The technique was first described in 1976 by Fernström and Johansson [1], and since then, advancements in surgical instruments and endourologic techniques have allowed the procedure to become widely accepted as a gold standard treatment for renal calculi [2-4].

According to the most recent updates of the European Urological Association guidelines, PCNL is the current treatment of choice for patients presenting with renal stones ≥2 cm in size [2,5]. The procedure involves creating a small percutaneous tract through the skin and into the kidney, allowing for direct access to the stone for fragmentation and removal [6].

Recent studies have demonstrated the efficacy and safety of PCNL, even in high-risk patient populations [7-9]. One retrospective analysis of over 2,200 PCNL procedures found no significant differences in surgical outcomes or complication rates between low-risk and high-risk patients, as defined by the American Society of Anesthesiologists (ASA) physical status classification system [10].

The surgical technique for PCNL involves several key steps. First, the patient is positioned appropriately, typically in the prone or supine position, so that the surgeon is afforded better access to the kidney and a wider area for percutaneous puncture and tract dilation [11]. The access point is then identified using fluoroscopic imaging, and a small puncture is made into the desired calyx. Once access is gained, the tract is serially dilated using Amplatz (Boston Scientific Corp., Natick, MA, USA), metal dilators, or balloon catheters to allow for the passage of the nephroscope. The stone is then fragmented using various intracorporeal lithotripters, such as ultrasonic or pneumatic devices, and the fragments are removed using suction or grasping forceps [2,6,10]. Following stone removal, a nephrostomy tube is left in place to allow for drainage and potential second-look procedures if necessary, although tubeless PCNL has its recognized benefits [6].

In Lebanon, the utilization of PCNL has been gradually increasing, as it offers a less invasive alternative to open surgery. As the prevalence of kidney stones continues to rise in Lebanon, it is essential to further investigate the safety and efficacy of PCNL in this specific context. Conducting comprehensive studies on the outcomes, complications, and long-term effects of this procedure in Lebanon would provide valuable data to guide clinical decision-making and improve patient care. Thus, the aim of this study is to evaluate the safety, efficacy, and clinical outcomes of PCNL in the Lebanese population, thereby contributing to the optimization of urolithiasis management strategies within the region.

## Materials and methods

Study population and ethical agreement

The study encompasses all patients who underwent PCNL at the Sheikh Ragheb Harb University Hospital (SRHUH) affiliated with Lebanese University from January 1, 2014, to April 1, 2024. Patients younger than 15 years or older than 80 years were excluded due to age-related differences in anatomy, perioperative care, and surgical instrumentation requirements, as well as the increased risk in these populations. The collected information included the following: patient admission number; patient admission date; age; medical history; surgical history; preoperative hematocrit; postoperative hematocrit; stone number; stone dimensions; stone location; diagnosis by non-contrast computed tomography (NCCT); stone-free status; kidney, ureter, and bladder X-ray (KUB) residual stone size; ultrasound residual stone size; operative time in minutes from initial ureteral catheter placement to the nephrostomy placement; hospital stay time in days; postoperative complications; postoperative blood transfusion; and number of surgeries needed.

The required data were collected from patients’ files after submitting the study protocol to the hospital’s Institutional Review Board (IRB) and obtaining its approval with the reference number IRB23RP19 on June 19, 2024.

Preoperative procedures

Forty-eight hours prior to the operation, the patient underwent preoperative laboratory testing, including complete blood count (CBC), serum creatinine analysis, prothrombin time (PT), partial thromboplastin time (PTT), international normalized ratio (INR), and urine analysis and culture. Patients with positive urine cultures (any growth within 48 hours) received targeted antibiotic therapy, and surgery was postponed until a sterile culture was confirmed. All patients with negative urine culture received intravenous first-generation cephalosporine, one hour before incision.

Applied surgical protocol

**Figure 1:**
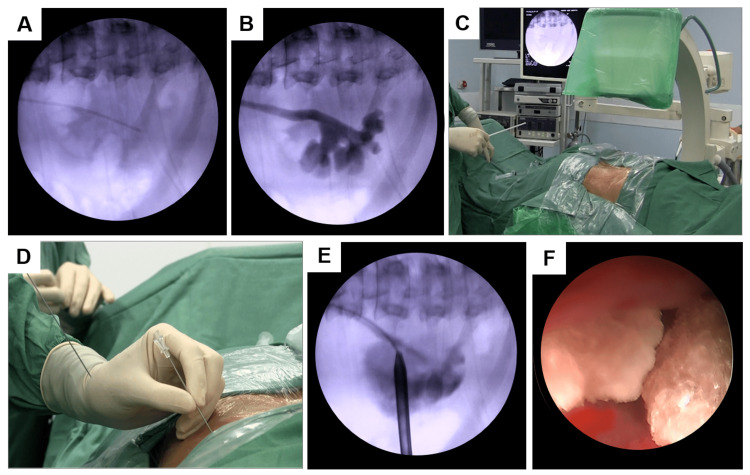
Applied surgical protocol of PCNL. (A) Fluoroscopic imaging showing insertion of a 6 Fr ureteral catheter into the renal collecting system. (B) Retrograde pyelography performed by injecting an iodine-based contrast medium under fluoroscopic guidance. (C) Patient positioned in the prone position, compatible with the C-arm fluoroscopy setup. (D) Chiba needle inserted into the calyx using the “eye-of-the-needle” technique, with confirmation of calyceal access by observing urine flow through the needle. (E) Fluoroscopic imaging of tract dilatation and Amplatz sheath placement into the calyx. The guidewire is correctly positioned, extending into the ureter. (F) Endoscopic view of renal calculi within the calyx using the nephroscope, prior to fragmentation and removal. PCNL: percutaneous nephrolithotomy

All procedures were performed under general anesthesia. All surgeries were performed by one senior urologist with a comparable experience level (having more than five years of independent endourology practice), supported by a dedicated anesthesia team trained in complex urologic procedures. The process begins with dorsal lithotomy to facilitate the retrograde insertion of a 6 Fr ureteral catheter into the renal collecting system (Figure 1A), allowing the opacification of the calyces during puncture by the injection of iodine-based contrast material under fluoroscopy (Figure 1B). The ureteral catheter is then fixed to a 14-18 Fr Foley catheter. Subsequently, the patient is repositioned into a prone position (Figure 1C), compatible with C-arm fluoroscopy.

After contrast injection into the collecting system using the ureteral catheter, a puncture is made using the “eye-of-the-needle” technique under fluoroscopy (Figure 1D). This technique is applied for the identification of the calyx to be punctured; the C-arm is positioned at a 30° angle toward the surgent. Subsequently, an 18 G access needle is carefully aligned so that the targeted calyx, the needle tip, and the needle hub form a straight line on the collimated image. This precise alignment results in an appearance reminiscent of the “eye of the needle” on the monitor. The C-arm is then moved away from the surgeon by a few degrees to determine the depth of the puncture. After confirming that the needle was correctly positioned within the calyx, the stylet is removed. The precise location of the needle is then verified using urine and contrast aspiration.

Subsequently, a 0.089 cm (0.035 in) hydrophilic guidewire is threaded through the puncture needle under fluoroscopic guidance to reach the ureter. Once the guidewire is appropriately placed, the puncture needle is withdrawn, and a 1 cm incision is made at the wire insertion site. The hydrophilic guide wire is replaced with a super stiff guide to allow dilation with Amplatz renal dilators to 26 Fr (Figure 1E). Throughout the last dilator, the working sheath of the nephroscope is placed, through which a 24 Fr rigid nephroscope (Karl Storz Endoskope, Tuttlingen, Germany) is used (Figure 1F). After that, stones are fragmented with pneumatic lithotripters (Swiss Lithoclast, EMS, Nyon, Switzerland) and removed using suction and forceps. By the end of stone fragmentation and removal, a double-j stent and an 18 Fr Pezzer catheter are placed in the working sheath and fixed to the skin.

Postoperative procedures

One day after the operation, a CBC test is performed, and the Foley catheter is removed. The nephrostomy tube is removed on the second postoperative day, and the patient is discharged home if his/her clinical status is assessed as stable.

One month after the operation, a KUB and an abdominal ultrasound and/or a CT scan of the abdomen and pelvis are performed to evaluate residual stones. Postoperative imaging is tailored based on stone radiopacity and intraoperative findings, using KUB and ultrasound for radiopaque stones, and resorting to NCCT for radiolucent stones or when residual fragments are suspected despite negative initial imaging. Stone-free status is defined as the absence of residual fragments or the presence of clinically insignificant fragments (<4 mm in diameter) by CT scan or KUB and ultrasound.

Statistical analysis

Data were collected using Microsoft Excel Version 2016 (Microsoft Corp., Redmond, WA, US). Statistical analyses and figure constructions were performed using the R program (version 4.3.2) (R Foundation for Statistical Computing, Vienna, Austria). For all statistical tests conducted in the study, a p-value of 0.05 or less was considered statistically significant.

## Results

All patients admitted to the Sheikh Ragheb Harb University Hospital between April 1, 2014, and April 1, 2024, who underwent PCNL operations, except those aged less than 15 years or over 80 years, were considered for this study, counting a total of 221 patients. The mean age of the study population was 50 ± 16 years, with 72% (n = 159) of the patients being men and 28% (n = 62) women (Table 1). The stone size mean was 3.1 ± 1.1 cm. The majority of stones were located in the pelvis (n = 94, 42.5%), which were the largest in size and the most complex stones; other stone locations included lower calyx (n = 46, 20.8%), lower calyx and pelvis (n = 37, 16.7%), and lower and middle calyx (n = 13, 5.8%). Most of the study individuals had single stones (n = 82, 37.1%), while 73 patients (33%) had multiple stones, and 66 patients (29.9 %) had staghorn stones (Table 1).

**Table 1:** Population characteristics and stone distribution.

Characteristic	Frequency and percentage
Number of patients	221
Males, n (%)	159 (72)
Age (years), mean ± SD	50 ± 16
Stone size (cm), mean ± SD	3.1 ± 1.1
Stone location, n (%)	
Pelvis	94 (42.5)
Lower calyx	46 (20.8)
Lower calyx & pelvis	37 (16.7)
Lower calyx & middle calyx	13 (5.8)
Others	31 (14)
Stone distribution, n (%)	
Single	82 (37.1)
Multiple	73 (33)
Staghorn	66 (29.9)

**Figure 2:**
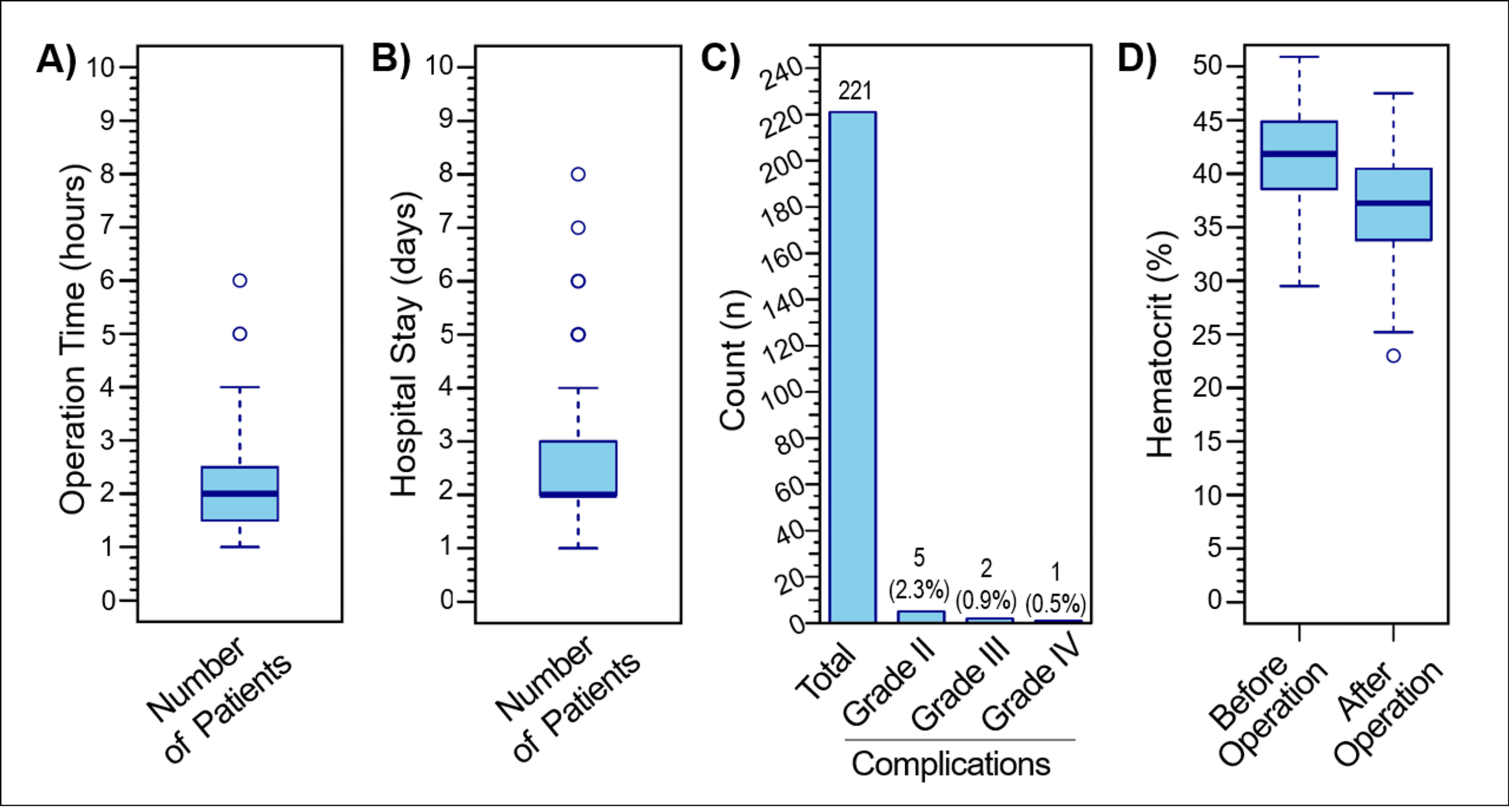
Safety indicators of PCNL procedures over the past 10 years. (A) The average time of PCNL operations over the 10-year interval was 2.1 ± 0.9 hours. (B) The average postoperative hospital stay was 2.7 ± 1.2 days. (C) Among the 221 operations, there were five grade II complications (2.3%), two grade III complications (0.9%), and one grade IV complication (0.5%) according to the Clavien-Dindo classification. (D) The comparison of patients’ hematocrit levels before and after the operation showed an average decrease of 4.7 ± 3.1% (paired t-test: t = 16.8, p < 0.0001). PCNL: percutaneous nephrolithotomy

The operation time mean was 2.1 ± 0.9 hours (Figure 2A), with only 15 cases exceeding three hours, and a maximum operation time of six hours for one case. The average postoperative hospital stay was 2.7 ± 1.2 days (Figure 2B). Among the 221 performed operations, five cases of grade II, two cases of grade III, and one case of grade VI complications, according to the Clavien-Dindo classification [12], were observed (Figure 2C). The five grade II complications involved persistent postoperative hematuria that required blood transfusion. Two grade III complication patients developed persistent hematuria that could not be managed conservatively, necessitating selective renal artery embolization by a vascular surgeon. These two patients were discharged home on the second day postembolization. The grade IV complication case developed a septic shock and was transferred to the Intensive Care Unit (ICU) for two days before being returned to the regular floor and discharged home after five days. During the 10-year study interval, no grade V complications, like nephrectomy or death, were observed. The patients' postoperative hematocrit showed a significant decrease (paired t-test: t = 16.8, p < 0.0001), with an average drop of 4.7% ± 3.1% (Figure 2D). These results indicate a high safety level of PCNL operations in the studied population, with only three major complications (grades III and IV) observed out of 221 operations.

**Figure 3:**
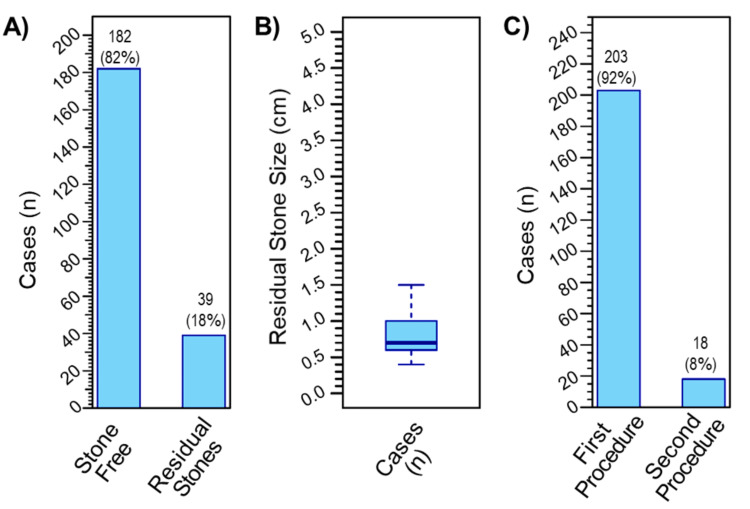
Outcomes of PCNL operations over the past 10 years. (A) Postoperative radiography showed that 82% of the performed PCNLs resulted in a stone-free status, while 18% of the cases had residual stones. (B) The mean of the residual stone size was 0.81 ± 0.32 cm. (C) Second intervention was needed in 8% of the performed PCNLs, while in 92% of the cases, the first procedure was sufficient. PCNL: percutaneous nephrolithotomy

At the level of PCNL outcomes, one-month postoperative radiography (KUB, abdominal ultrasound, and/or abdomen and pelvis CT scan) revealed that out of the 221 cases, 82.35% (n = 182) of the operations resulted in stone-free status, while residual stones were observed in 17.65% (n = 39) of the study population (Figure 3A). The mean of the residual stone size was 0.81 ± 0.32 cm (Figure 3B). The majority of cases (91.9%, n = 203) did not need a second intervention, while only 8.1% (n = 18) needed a second intervention that was mainly performed by flexible ureteroscopy or second PCNL (Figure 3C).

**Figure 4:**
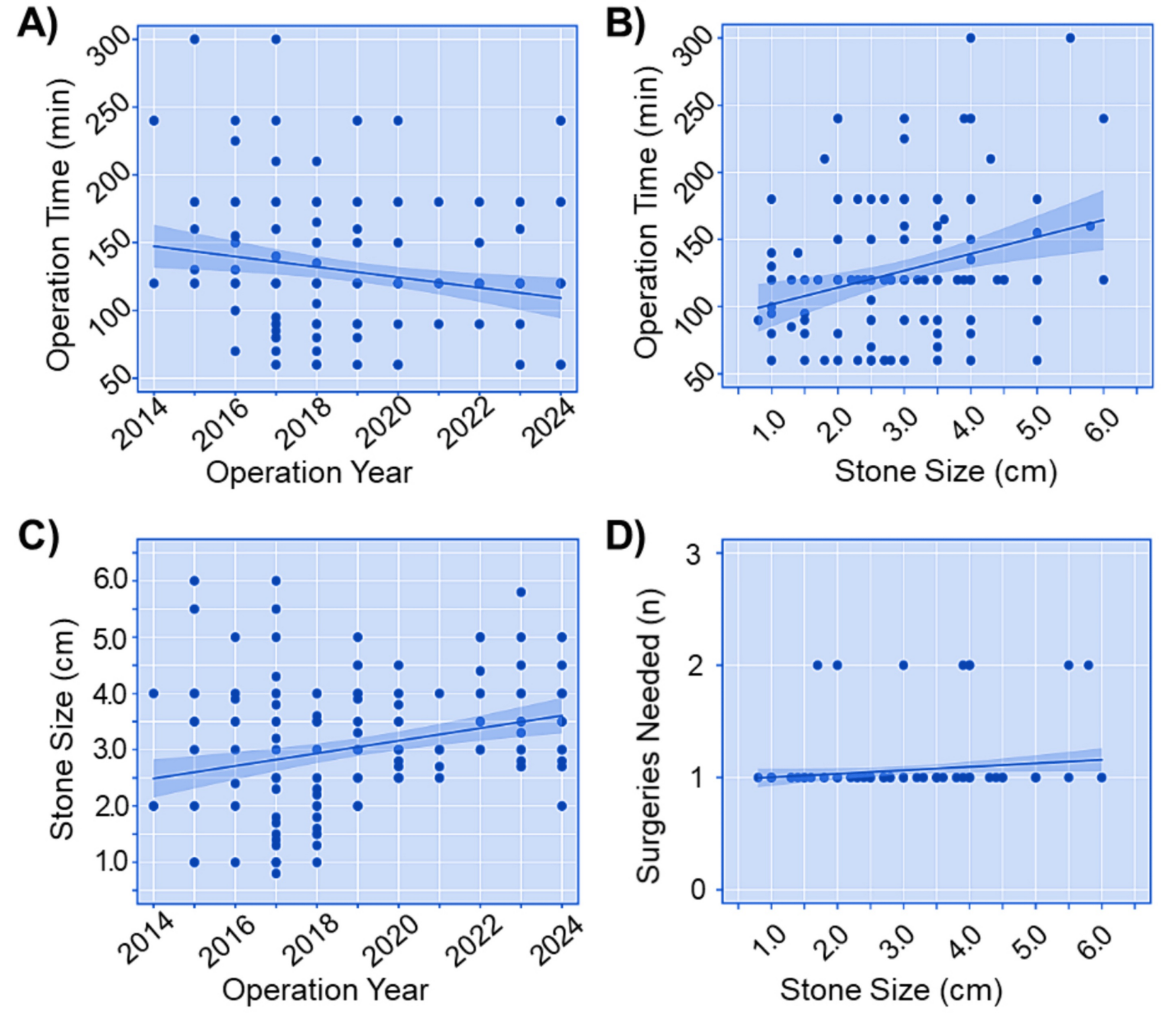
Regression analysis of factors influencing PCNL safety and efficacy over 10 years. (A) The relationship between operation time and admission date showed a significant negative trend (linear regression: β = -3.82, SE = 1.39, t-value = -2.75, p-value = 0.006). The shaded area represents the 95% confidence interval. (B) A significant positive correlation was observed between stone size and operation time (linear regression: β = 12.6, SE = 3.6, t-value = 3.5, p-value = 0.006). (C) A significant positive correlation was observed between stone size and operation year (linear regression: β = 0.1, SE = 0.03, t-value = -3.8, p-value = 0.0001). (D) No significant correlation was observed between the number of surgeries needed for stone clearance and stone size (linear regression: β = 0.03, SE = 0.2, t-value = 1.9, p-value = 0.06). β: estimate; SE: standard error; PCNL: percutaneous nephrolithotomy

Based on the data collected over the past 10 years, a primary analysis was conducted to determine the key factors affecting PCNL safety and efficacy. A linear regression analysis demonstrated a significant association between operation time and admission date (linear regression: β = -3.82, standard error (SE) = 1.39, t-value = -2.75, p-value = 0.006), highlighting the substantial impact of the healthcare team's growing experience on reducing operation time (Figure 4A). Moreover, a significant positive correlation (linear regression: β = 12.6, SE = 3.6, t-value = 3.5, p-value = 0.006) was observed between stone size and operation time (Figure 4B), which is an expected result, as larger and more complex stones require additional time and effort for clearance. To ensure that the results in Figure 4A are not influenced by a potential decrease in stone size over the years, we examined the correlation between stone size and the year of operation. The results demonstrated a significant positive correlation (linear regression: β = 0.1, SE = 0.03, t-value = -3.8, p-value = 0.0001) between stone size and operation year (Figure 4C). Finally, our analysis did not find a significant correlation (linear regression: β = 0.03, SE = 0.2, t-value = 1.9, p-value = 0.06) between the number of surgeries needed for stone clearance and stone size (Figure 4D). Overall, these findings indicate that the experience of healthcare professionals and stone size are critical factors that influence the safety and efficacy of PCNL operations.

## Discussion

PCNL is currently considered the therapeutic method of choice for the treatment of large (>3 cm diameter) and complex renal stones [13], with high safety [14] and efficacy rates reported in many healthcare centers worldwide [7]. In this study, we aimed to share our 10-year experience in this field with the healthcare community, focusing primarily on the variables that reflect the safety and efficacy of the applied protocols and procedures.

To assess the safety and efficacy of our procedures, we collected and analyzed a set of variables that have been recognized as essential indicators in previously published studies. One of the important safety indicators is the average operation time, as longer surgery periods increase the risk of complications such as bleeding, infections, and anesthesia-related issues. The average operation time in our study was 2.1 hours, which is comparable to the average times reported by Stojanoski et al. (1.98 hours) [11] and Kurtulus et al. (2.2 hours) [13]. However, Tseng et al. reported a longer average operation time of 3.05 hours [14]. Investigating the cause of this discrepancy, we observed that stone characteristics, such as average stone size, location, and the percentage of staghorn stones, in both studies differ significantly. Moreover, the target populations’ sizes varied widely between the two studies, with 221 cases considered in our study compared to 76 patients in the study of Tseng et al. [14]. While Tseng et al. did not find a significant correlation between stone size and operation time, our analysis revealed a strong positive correlation. Further multicenter studies could help clarify these differences and establish a benchmark operation time that accounts for such variables.

Another key safety indicator is the rate and severity of perioperative and postoperative complications. We observed a total of eight complications among the 221 operations performed (3.6%), including five grade II, two grade III, and one grade IV complication according to the Clavien-Dindo classification [12]. This complication rate is considerably lower than the 20%-30% range reported in previous studies [14,15]. Additionally, longer postoperative hospital stays are often associated with complications such as infections or significant bleeding. In our study, the average postoperative hospital stay was 2.7 ± 1.2 days (range: 1-8 days), which is shorter than the 4-6 days reported in other studies [14,16,17]. These findings underscore the importance of the collective expertise of the operating team, the proficiency of the anesthesia team, and effective infection control measures.

The second critical aspect in our study, after safety, is the efficacy of the operation. High-efficacy surgeries result in a high stone-free rate with a minimal need for additional procedures. In our data, the stone-free rate was over 82%, with the mean residual stone size being approximately 0.8 cm. These results align with the stone-free rates reported by El-Nahas et al., Stojanoski et al., and Ali et al., which ranged between 78% and 81% [10,11,18]. Additionally, only 8% of the PCNL procedures in our study required a second operation, compared to 30% in El-Nahas et al.’s study [10] and 13.71% in Ali et al.’s study [18].

The achievement of stone-free status, and thus reducing the need for a second operation, is influenced by several factors. These factors can be categorized into three groups: stone-related (size, anatomical location, and composition), patient-related (renal anatomy, obesity, and previous surgeries), and surgical-related (creation of an optimal access tract, complete stone fragmentation) factors. Identifying and managing these factors, in addition to supporting the surgical team with advanced imaging and fragmentation technologies, improves the stone-free rate and reduces the need for additional procedures.

Overall, reflecting on our experience, we believe that several factors contributed to the successful outcomes of our PCNL procedures, the most important of which is the surgical team's experience, combined with the advanced technologies used for imaging and stone fragmentation. Furthermore, adherence to well-structured preoperative assessments allowed us to individualize patient care based on the stone- and patient-related factors.

Despite the strengths of our 10-year retrospective analysis, this study is not without limitations. First, the retrospective design inherently carries the risk of incomplete documentation, particularly regarding follow-up imaging and long-term outcomes. Second, while we included a relatively large sample size for a single-center study, the results may not be generalizable to other institutions due to variations in surgical techniques, equipment availability, and patient demographics. Third, our definition of stone-free status was based on imaging performed one month postoperatively, which may not accurately reflect longer-term outcomes or recurrence rates. Moreover, the lack of data on stone composition and patient comorbidities limited our ability to explore additional predictors of surgical success and complications. Future prospective multicenter studies incorporating standardized imaging protocols and patient-centered outcomes would be valuable in addressing these limitations and enhancing the generalizability of our findings.

Finally, despite the significant advances made in PCNL operations globally, there is still no universally accepted benchmark for safety and efficacy that medical and quality departments can use to evaluate and enhance their practices. Establishing such benchmarks depending on the documented last 20 years of global experience is extremely valuable in this field. In addition, future research should focus on standardizing safety and efficacy criteria to allow for more consistent evaluation across different healthcare settings.

## Conclusions

Our decade of experience with PCNL attests to its security and efficacy in treating large and complex renal stones, underscoring the value of surgical expertise, structured preoperative assessment, and emerging technology. Yet, the absence of standardized criteria for PCNL procedures testifies to the necessity of regional multicentric studies and uniform protocols to enhance clinical practice and deliver similar, patient-oriented outcomes.
